# Intra-topic latency as an automated behavioral marker of treatment response in autism spectrum disorder

**DOI:** 10.1038/s41598-022-07299-w

**Published:** 2022-02-28

**Authors:** Elizabeth P. McKernan, Manoj Kumar, Adriana Di Martino, Lisa Shulman, Alexander Kolevzon, Catherine Lord, Shrikanth Narayanan, So Hyun Kim

**Affiliations:** 1 Department of Psychiatry, Weill Cornell Medicine, New York, NY USA; 2 Ming Hsieh Department of Electrical and Computer Engineering, University of Southern California, Los Angeles, CA USA; 3 Child Mind Institute, New York, NY USA; 4 Department of Pediatrics, Albert Einstein College of Medicine, New York, NY USA; 5 Seaver Autism Center for Research and Treatment, Department of Psychiatry, Icahn School of Medicine at Mount Sinai, New York, NY USA; 6 Semel Institute for Neuroscience and Human Behavior, David Geffen School of Medicine, University of California Los Angeles, Los Angeles, CA USA

**Keywords:** Neurodevelopmental disorders, Outcomes research

## Abstract

Data science advances in behavioral signal processing and machine learning hold the promise to automatically quantify clinically meaningful behaviors that can be applied to a large amount of data. The objective of this study was to identify an automated behavioral marker of treatment response in social communication in children with autism spectrum disorder (ASD). First, using an automated computational method, we successfully derived the amount of time it took for a child with ASD and an adult social partner (*N* pairs = 210) to respond to each other while they were engaged in conversation bits (“latency”) using recordings of brief, natural social interactions. Then, we measured changes in latency at pre- and post-interventions. Children with ASD who were receiving interventions showed significantly larger reduction in latency compared to those who were not receiving interventions. There was also a significant group difference in the changes in latency for adult social partners. Results suggest that the automated measure of latency derived from natural social interactions is a scalable and objective method to quantify treatment response in children with ASD.

## Introduction

Automatically-derived, objective audio–video measures of behavior can complement and enhance traditional manual behavioral coding, providing an alternative way to identify treatment response^1,2^. Signal processing and machine learning techniques have been applied to study social interactions of children with autism spectrum disorder (ASD). These efforts have yielded a number of findings relevant to treatment outcome, including the identification of acoustic-prosodic properties that can be measured objectively and are related to autism symptom severity^3,4^.

The goal of the present study was to assess response to treatment as measured by automatically extracted conversational features from a brief, natural social interaction (i.e., Brief Observation of Social Communication Change [BOSCC]^5^). To date, outcome measures for ASD have largely consisted of study-specific, manually coded behavioral observations or parent-, therapist- or self-reports^6^; many of these measures do not directly target core autism symptoms such as social communication impairments even though understanding whether core autism symptoms are effectively impacted by treatments is the key to evaluate their efficacy^7–9^. It has also been shown that relying solely on caregiver-, therapist-, and/or self-reports may introduce measurement error^10,11^. This is due to a variety of factors including bias and unblinding. Thus, unbiased automated coding can complement traditional behavioral measures by humans. The detection of treatment response using an automated method also has high scalability to process a large amount of data, allowing us to overcome the constraints in time and efforts.

Previous outcome measures for interventions focused on improving communicative or conversational skills have included task analyses^12^, Likert scales^13^, and probe data^14^, none of which incorporated computational methods of data interpretation using automatic signal and information processing techniques. The automatically derived conversation behaviors have the potential to transform the field by providing an objective and scalable measure of treatment response that augments human judgment and enhances traditional manual coding. Previous work examined automated detection, modeling, and analyses of behaviors of children with ASD interacting with a social partner in the context of diagnostic evaluations. Results suggested that children with greater autism symptom severity spoke less, took longer to respond (longer latency), as well as used personal pronouns and affect language less often^4^. Expanding on these findings, for the first time in the field, we used automatically extracted conversational features, specifically latency, or the time it takes for social partners to respond to each other, to measure treatment response in children with ASD. Automated coding of latency provides a highly specific temporal resolution which augments human coding, given that these features are impossible for human coders to measure with high precision.

### Latency

Previous work focused on latency demonstrated that children with ASD often take longer to respond within a conversation. For instance, children with ASD were found to take 23.7% longer to respond than typically developing (TD) children in back-and-forth conversation, and 34.4% longer to respond to questions than TD children^15^. Another study^16^ showed that response latency positively correlated with the overall Autism Diagnostic Observation Schedule (ADOS^17^) severity score and the social communication ADOS severity score, pointing to the utility of using “intra-topic” latency (the time it takes to respond to a social partner within a conversation topic). Therefore, in our study, we hypothesized that latency would serve as an effective treatment outcome measure for children with ASD. In addition, recent work suggested that adult social partners while interacting with a child with ASD adjust their own behaviors; thus, their speech properties are more predictive of the child’s social communication impairments than the child’s own atypical speech patterns^18^. Based on this, we hypothesized that dynamic interactions between dyads would lead to changes in latency in both children with ASD and their social partners over the course of treatment.

## Results

### Association between latency and baseline clinical features

Bivariate correlations were conducted to determine whether there was a significant association between latency and clinical measures of autism severity (ADOS-2 Calibrated Severity Score–Social Affect [CSS SA]) and cognitive functioning (nonverbal IQ [NVIQ], verbal IQ [VIQ]). Child and examiner latency variables were significantly positively correlated with each other. Child intra-topic latency was significantly positively correlated with the ADOS-2 CSS SA. On the other hand, neither of the latency variables were significantly correlated with VIQ nor NVIQ. Bivariate correlations are presented in Table 1.

**Table 1:** Bivariate correlations between clinical measures and intra-topic latency.

	1	2	3	4	5	6	7	8
1. VIQ	1							
2. NVIQ	0.84**	1						
3. Expressive language level	− 0.09	− 0.09	1					
4. CSS SA	− 0.07	− 0.01	0.05	1				
5. CSS RRB	− 0.03	0.02	− 0.01	0.25**	1			
6. Age	− 0.02	− 0.01	0.36**	0.12*	− 0.01	1		
7. Child latency intra-topics	− 0.03	− 0.04	− 0.05	0.24**	− 0.04	− 0.02	1	
8. Examiner latency intra-topics	− 0.01	− 0.03	− 0.02	0.08	0.04	0.04	0.36**	1

N = 333–350.

*p < 0.05, **p < 0.01.

### Changes in latency over the course of interventions

**Figure 1:**
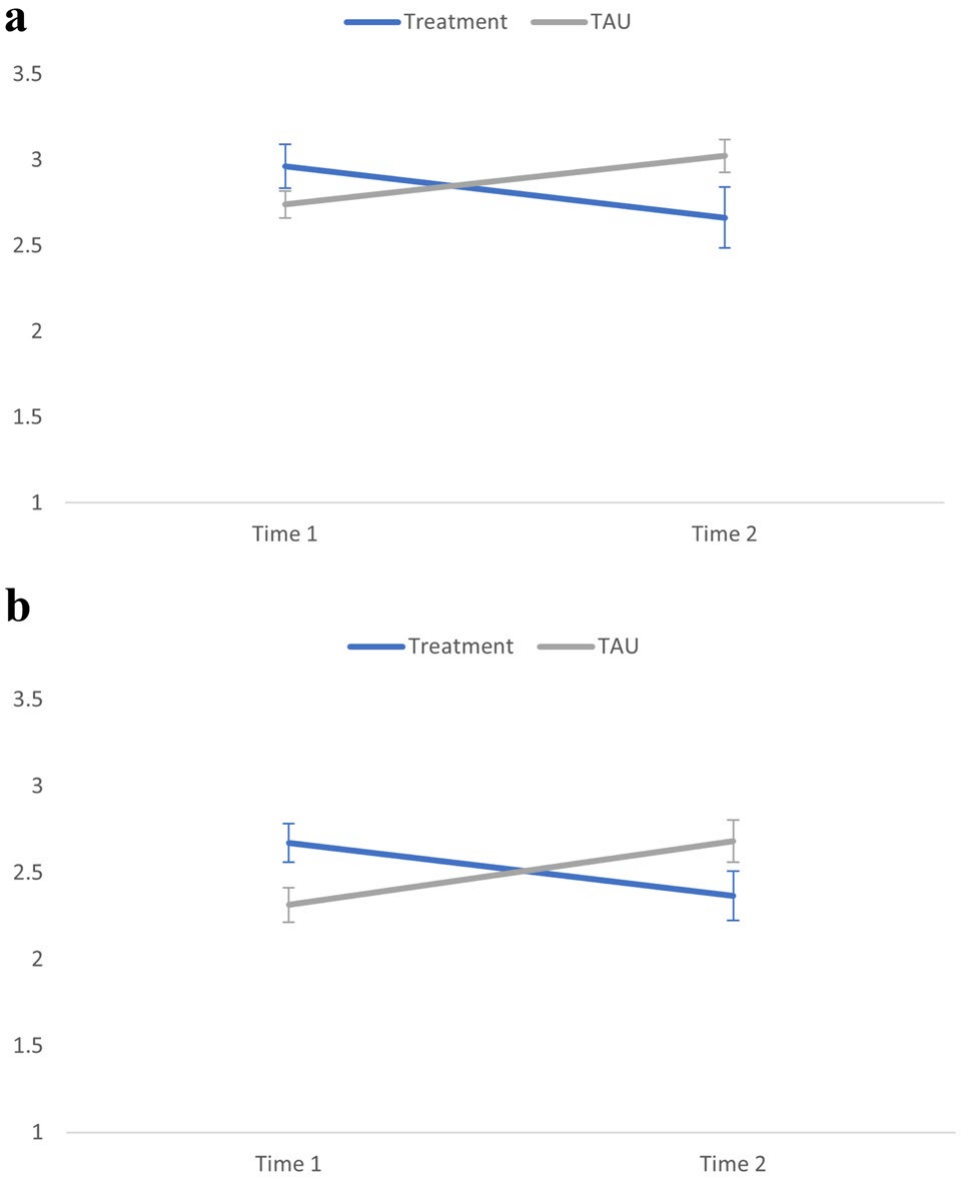
(**a**) Estimated means for child intra-topic latency. Error bars depict ± 2 standard errors. (**b**) Estimated means for examiner intra-topic latency. Error bars depict ± 2 standard errors.

There was a significant interaction between time and treatment condition for both child intra-topic latency as well as examiner intra-topic latency (see Table 2). As depicted in Fig. 1a, the intra-topic latency of children who received treatment significantly decreased from pre- to post-assessment (*t*[172] = 2.05, *p* = 0.042, *d* = 0.16; *M* = 2.94, *SD* = 1.77 at Pre-intervention, *M* = 2.69, *SD* = 1.33 at Post-intervention), whereas intra-topic latency significantly increased over time for children in the “treatment as usual” (TAU) condition (*t*([175] = − 3.46, *p* = 0.001, *d* = − 0.28; *M* = 2.63, *SD* = 1.21 at Pre-intervention, *M* = 3.05, *SD* = 1.79 at Post-intervention). As shown in Fig. 1b, examiners’ intra-topic latency was stable from pre- to post-assessment when interacting with children who received treatments (*t*[172] = 1.82, *p* = 0.071, *d* = 0.07; *M* = 2.54, *SD* = 2.49 at Pre-intervention, *M* = 2.38, *SD* = 2.38 at Post-intervention) but significantly increased over time when interacting with children in the TAU group (*t*[175] = − 2.07, *p* = 0.04, *d* = − 0.17; *M* = 2.29, *SD* = 1.36 at Pre-intervention, *M* = 2.55, *SD* = 1.64 at Post-intervention). Across groups, the effects of baseline expressive language level and autism symptom severity on latency were also significant such that children with higher expressive language levels and those with lower symptom severity had shorter intra-topic latency.

**Table 2:** Generalized linear mixed models of time and treatment effects for intra-topic latency.

Outcome variable	Predictors	*B*	*df*	*F*	Significance
Child intra-topic latency	Overall model		[17, 313]	2.53	0.001**
	Time	0.37	[1, 313]	0.001	0.969
	Treatment condition	0.52	[1, 313]	0.732	0.393
	Time * treatment condition	− 0.73	[1, 313]	5.32	0.022*
	**Expressive language**	4.74	[4, 313]	4.93	0.001**
	Level = 5	− 0.24			
	Level = 6	0.08			
	Level = 7	− 0.35			
	NVIQ	< 0.001	[1, 313]	0.210	0.647
	**CSS SA**	− 1.28	[7, 313]	2.12	0.041*
	CSS SA = 4	− 1.19			
	CSS SA = 5	− 1.17			
	CSS SA = 6	− 0.94			
	CSS SA = 7	− 0.64			
	CSS SA = 8	− 0.52			
	CSS SA = 9	− 0.38			
	Age	− 0.002	[1, 313]	0.703	0.402
	Gender	− 0.07	[1, 313]	0.079	0.779
Examiner intra-topic latency	Overall model		[17, 313]	1.05	0.408
	Time	0.29	[1, 313]	0.087	0.768
	Treatment condition	0.42	[1, 313]	0.080	0.777
	Time * treatment condition	− 0.67	[1, 313]	4.40	0.037*
	**Expressive language**	2.52	[4, 313]	1.02	0.396
	Level = 5	0.43			
	Level = 6	− 0.15			
	Level = 7	− 0.49			
	NVIQ	− 0.001	[1, 313]	0.320	0.572
	**CSS SA**	− 1.19	[7, 313]	1.14	0.339
	CSS SA = 4	− 0.71			
	CSS SA = 5	0.51			
	CSS SA = 6	− 1.07			
	CSS SA = 7	− 0.56			
	CSS SA = 8	− 0.80			
	CSS SA = 9	− 0.64			
	Age	< 0.001	[1, 313]	0.002	0.962
	Gender	− 0.28	[1, 313]	0.427	0.514

*NVIQ* nonverbal IQ, *CSS SA* calibrated severity score-social affect.

*p < 0.05, **p < 0.001.

When treatment duration, site, and the interaction between treatment condition and site were controlled within the mixed models, treatment duration (*F*[1, 215] = 0.008, *p* = 0.929 for child latency; *F*[1, 215] = 0.118, *p* = 0.732 for examiner latency) and the interaction between treatment condition and site (*F*[1, 215] = 0.23, *p* = 0.631 for child latency; *F*[1, 215] = 0.431, *p* = 0.512 for examiner latency) were not significant, but there was a significant effect of site for child latency (*F*[3, 215] = 2.96, *p* = 0.033 for child latency; *F*[3, 215] = 1.39, *p* = 0.245 for examiner latency); however, all of the significant results on the interaction effect between the time and treatment condition described above remained the same. Post-hoc pairwise comparisons with Bonferroni correction revealed that the child latency mean for the Montefiore site was significantly lower than that of the ISMMS and CADB sites at pre-intervention, and significantly lower than that of the CADB site at post-intervention (all *p* < 0.05).

We also calculated change scores by subtracting pre-assessment values from post-assessment values of both child and examiner intra-topic latency. Change scores for child intra-topic latency were significantly different between treatment and TAU groups (*t*[347] = 4.05, *p* < 0.001, *d* = 0.43). Similarly, change scores for examiner intra-topic latency were also significantly different between treatment and TAU groups (*t*[347] = 2.8, *p* = 0.005, *d* = 0.30).

Finally, regression analyses predicting Post-Intervention latency while controlling for the Pre-Intervention latency as well as NVIQ, autism symptom severity, age, expressive language level, and gender revealed differences between the treatment groups (child latency, *B* = − 0.530, *p* = 0.001 for the treatment condition, *R*^2^ = 0.26, *F*[7, 325] = 16.49, *p* < 0.001 for the overall model; examiner latency, *B* = − 0.405, *p* = 0.008 for the treatment condition, *R*^2^ = 0.58, *F*[7, 325] = 63.32, *p* < 0.001 for the overall model), with the treatment group showing significantly shorter latency compared to the TAU group for both children and examiners.

## Discussion

The present study used a novel automated measure to identify a behavioral marker of treatment response, specifically intra-topic latency, within a matched sample of children who did and did not receive focused short-term treatments to improve social communication symptoms. The finding of a significant interaction between time and treatment condition for the automated measure of child intra-topic latency reveals that children showed significantly larger reduction in latency over the course of a relatively short-term (i.e., 3 to 4 months’ duration) intervention designed to improve social skills. At the same time, there was also a significant interaction between time and treatment condition for examiner intra-topic latency. Specifically, examiners interacting with children who were not receiving interventions showed increased latency over time, whereas those interacting with children who were receiving intervention did not show any difference in latency from pre- to post- intervention. These results imply a reciprocal relation between the behavior of the child and the adult in the context of a brief social interaction.

Previous work by Bone et al.^3,18^ using conversational samples taken from the ADOS found that the psychologist’s behavior was reflective of the child’s social-communicative behavior, in that the psychologist’s prosody was more predictive of the child’s ASD severity than the child’s own prosody. Similarly, in a follow-up study, Bone et al.^4^ found in cross-sectional studies that as ASD severity increases, psychologists varied their speech and language strategies in attempts to engage children in social interaction. These results, along with those of the present study on observed changes in latency of both children with ASD and their social partners, fit well within the literature on Communication Accommodation Theory^19,20^. This theory views interpersonal conversation as a dynamic adaptive exchange in which a speaker’s speech signal features are tailored to their conversational partner to maximize intelligibility and efficiency. Given the potential for interpersonal synchrony to be a marker of the quality of social interactions^21–23^ as well as a potential avenue for intervention^24,25^ among ASD populations, intra-topic latency could provide an objective and quantifiable measure of conversational coordination and synchrony.

Additionally, child intra-topic latency was significantly correlated with the ADOS-2 Calibrated Severity Score, also indicating that these features may be useful indicators of clinical measures of overall autism severity. Importantly, neither latency variable was correlated with IQ, suggesting that these features may be separable from cognitive functioning in verbal children with ASD.

There are a few limitations of the study. First, this study was not a randomized controlled trial (RCT). Participants in the treatment and TAU conditions were not evenly represented across sites, with the Center for Autism and the Developing Brain site having a greater number of participants in the TAU condition than would be expected. In the future, we hope to extend our work to validate the automated method in a more controlled setting. We also observed a site effect, although the results remained the same when the effect of site was controlled for. An RCT design across sites will help address the potential confound further. In addition, the lack of extant literature regarding the distribution of intra-topic latency within non-ASD populations across different ages precludes our ability to determine normative values of this measure. Comparison of this clinical feature across typical and atypical populations will reveal greater insight into what might constitute clinically abnormal latency values. Our work was also limited to children and adolescents who were verbal, and thus the results cannot be generalized to verbal adults or minimally verbal individuals. Finally, it is important to note that 65 out of 140 dyads (46.4%) had different social partners at pre- and post-interventions. Importantly, there was no significant difference between the treatment and TAU groups in the number of participants who had the same examiner at both time points, χ^2^(1, n = 210) = 0.083, *p* = 0.773. When the analyses were limited to the dyads who had same examiners at pre- and post-interventions (n = 75 out of 140 dyads; 53.6%), the interaction effect between time and treatment condition remained marginally significant, potentially due to limited statistical power (*F*[1, 183] = 2.94, *p* = 0.088 for child latency; *F*[1, 183] = 2.83, *p* = 0.095 for examiner latency).

## Conclusion

The present study was the first to apply signal processing and machine learning techniques to derive an automated measure of treatment response in children with ASD. Specifically, the results demonstrate that automatically derived intra-topic latency can serve as an objective and scalable treatment response measure in youth with ASD who have received social skills training or other forms of interventions. Given the current lack of standard autism-specific treatment response measures that are sensitive enough to capture change^7^, our results may push the field to implement automated measures of audio–video signals, which have the potential to augment human perception and judgment^1^. To this end, we leveraged the BOSCC recordings intended to provide the standardized settings of natural social interactions between a child with ASD and a social partner for automated analyses of audio and video data. Our current findings suggest that intra-topic latency in youth with ASD has potential as an indicator of treatment response. Forthcoming research would do well to use latency and other automatically extracted conversational features to examine treatment effects in the context of a broader range of ASD interventions and clinical trials.

## Methods

### Participants

Data were obtained from a study of the sensitivity and reliability of new outcome measures in capturing treatment responses for children with ASD who were receiving specific interventions at four autism clinics in university-based centers in the New York metropolitan area, compared to a “treatment as usual” (TAU) group who did not receive any interventions at the clinics. Thus, this was not a randomized clinical trial. Participants included 210 children with ASD between the ages of 4 and 17 years from Center for Autism and the Developing Brain (CADB) at Weill Cornell Medical College (n = 62), Child Study Center at the New York University Langone Medical Center (NYU; n = 62), the Seaver Autism Center for Research and Treatment at the Icahn School of Medicine at Mount Sinai (ISMMS; n = 37), and Montefiore Medical Center/Albert Einstein College of Medicine (Montefiore; n = 49). All participants had phrase speech (i.e., flexible 3- to 4-word combinations; n = 22) or verbally fluent language (i.e., use complex sentences and can speak about the past and future; n = 188). Among these children, half of them (n = 105) were participating in social groups or other forms of treatments (see below) at the four university-based centers listed above. Specifically, participants at CADB received individual and group behavior interventions focused on improving social communication in children with ASD, such as the Program for the Education and Enrichment of Relational Skills (PEERS^®^^26^) and the Secret Agent Society (SAS)^27^ as well as a clinical trial testing the use of intranasal oxytocin for the treatment of autism symptoms^28^. Participants at NYU received parent-mediated social skills interventions in a group setting, including PEERS^®^^26^ and the Children’s Friendship Program (CFP)^29,30^. Participants at ISMMS took part in a clinical trial testing the use of intranasal oxytocin for the treatment of autism symptoms, as well as family peer advocate services (FPA)^31,32^. Participants at Montefiore received individual and group social skills interventions using the Social Thinking curriculum^33,34^. Children were asked to delay medication changes, when possible. Mean treatment duration was 3.25 months (SD = 2.05 months, range = 1–16 months).

In addition, the other 105 children with ASD from the same sites who were not actively receiving these or other similar treatments served as a “treatment as usual” (TAU) group. Participants in the TAU group included children who received diagnostic assessments but no treatment, as well as those on waiting lists for services, at the four sites. This comparison group included children of equivalent age, IQ, autism symptom severity, gender, race, ethnicity, and maternal education level (see Table 3). We conducted a chi-square test of independence to examine the relation between treatment condition and site. The association was significant, χ^2^(3, N = 210) = 8.58, *p* = 0.036, with the CADB site having a larger proportion of TAU cases compared to treatment cases. Specifically, observed count for the TAU condition at CADB was 40, versus an observed count of 22 in the treatment group (expected count of 31 for both treatment and TAU groups).

**Table 3:** Baseline demographic characteristics.

	Treatment (*n* = 105) M (SD)	Treatment-as-usual (*n* = 105) M (SD)	*t* statistic
Age (months)	113.4 (36.8)	108.7 (38.6)	− 0.885
**Cognitive skills**			
VIQ	99.9 (19.1)	99.4 (18.8)	− 0.206
NVIQ	100.7 (16.7)	101.7 (18.1)	0.390
**ADOS-2**			
CSS SA	7.5 (1.9)	7.7 (1.7)	0.739
CSS RRB	6.8 (2.4)	6.4 (2.7)	− 1.03

	Treatment *n* (%)	Treatment-as-usual *n* (%)	*Χ*^2^ statistic
**Sex**			0.147
Males	90 (85.7)	88 (83.8)	
**Race**			5.36
White	58 (55.2)	51 (48.6)	
African American	10 (9.5)	16 (15.2)	
Asian/Pacific Islander	7 (6.7)	5 (4.8)	
American Indian	2 (1.9)	1 (1.0)	
Biracial	9 (8.6)	9 (8.6)	
Other/unspecified	16 (15.3)	14 (13.3)	
**Ethnicity**			0.934
Hispanic	30 (28.6)	22 (21.0)	
**Maternal education**			5.50
High school diploma	4 (3.8)	4 (3.8)	
Some college/vocational	12 (11.4)	5 (4.8)	
Associate degree	6 (5.7)	5 (4.8)	
Four-year college degree	22 (21.0)	11 (10.5)	
Graduate/professional degree	26 (24.8)	21 (20.0)	

Race was not reported for 3 participants in the treatment group and 9 participants in the treatment-as-usual group. Ethnicity was not reported for 22 participants in the treatment group and 29 participants in the treatment-as-usual group. Maternal education level was not available for 30 participants in the treatment group and 54 participants in the treatment-as-usual group.

### Ethics

This study was approved by the Institutional Review Boards of Weill Cornell Medicine (#1507016398), New York University Langone Medical Center (#i15-00168 and #i15-00804), the Icahn School of Medicine at Mount Sinai (#15-0325), and the Albert Einstein College of Medicine (#2015-5508). This work was carried out in accordance with the ethical standards of the institutions named above and with the Declaration of Helsinki. Written informed consent was obtained from parents of all participants.

### Measures

#### BOSCC

The Brief Observation of Social Communication Change (BOSCC)^5^ is a measure of treatment response for social communication behaviors. The BOSCC consists of specific items designed to identify changes in social communication behaviors over relatively short periods of time by quantifying information about the behaviors’ quality and frequency. The BOSCC was initially developed by expanding and adding to ADOS-2 items, but it has now been substantially revised. It is coded from a videotaped observation of a child during a naturalistic social and play interaction. The BOSCC has also been applied to segments of the ADOS in a group of young, minimally verbal children with ASD, and was found to be more sensitive in monitoring changes over the course of treatment as compared to the ADOS Calibrated Severity Score^35^. This study was an early component of a larger effort to develop behavioral coding schemes across language and age levels that is still underway. Tasks from this initial version of the BOSCC for verbal children provide optimal opportunities to obtain language samples of conversation between a child with ASD and an adult social partner within the 12-min, semi-structured, natural interactions. The social partners (examiners) were blind to the treatment condition of the children. The tasks consisted of 4 min of play with various age-appropriate materials (e.g., pinball game, puzzles, transformers, picture books, scratch art, foreign money, conversational cards), followed by 2 min of conversation without any materials present, and another 4 min of play with a different set of materials and 2 min of conversation. The BOSCC was administered prior to treatment entry and again following treatment completion for children in the treatment group. Children in the TAU group were administered the BOSCC before and after a time determined by yoking by treatment length.

#### Social communication symptom levels

For descriptive purposes, research reliable clinicians administered and scored the Autism Diagnostic Observation Schedule, 2nd Edition (ADOS-2)^17^ for all children prior to treatment entry. The ADOS-2 provides an overall Calibrated Severity Score for social communication symptoms under the Social Affect domain (CSS SA)^36^. The overall level of language item from the ADOS-2 was also used to quantify the language levels, following the new metric that has been validated in a large sample across different ADOS-2 Modules^37^. Higher scores indicate higher language levels (e.g., a score of 5 = regular use of utterances with two or more words; a score of 6 = speech is mostly utterances of at least three words, but without complex language; a score of 7 = some relatively complex speech but with recurrent grammatical errors not associated with use of dialect; a score of 8 = uses sentences in a largely correct fashion). Of the 210 participants, 22 were administered ADOS-2 Module 2, 185 were administered Module 3, and 3 were administered Module 4 (for adolescents ages 16–17 years).

#### Cognitive levels

For descriptive purposes, examiners blind to the purposes of the study administered cognitive assessments to participants at baseline, including the Differential Ability Scales, Second Edition (DAS-II)^38^, Stanford Binet Intelligence Scales, Fifth Edition (SB-5)^39^, Wechsler Preschool and Primary Scale of Intelligence, Fourth Edition (WPPSI-IV)^40^, Wechsler Intelligence Scales for Children, Fifth Edition (WISC-V)^41^, Wechsler Adult Intelligence Scale, Fourth Edition (WAIS-IV)^42^, and Wechsler Abbreviated Scale of Intelligence, Second Edition (WASI-II)^43^. Mean verbal IQ and nonverbal IQ values are reported in Table 3.

### Audio data collection

A portable audio–video recording set-up was standardized across all four sites to eliminate unwanted differences in data due to site-specific biases. Specifically, video of the child’s face, movements of participants, and the overall interaction was captured from one high-definition camcorder mounted in the corner of the clinical space. Audio was recorded from four high-quality microphones: one far-field shotgun microphone, mounted on the camcorder, recorded the voices of participants along with any ambient noise. Two lapel/lavalier microphones, one attached to the clothing of the child and the other to the examiner, recorded near-field high quality speech signals of each participant. A boundary/surface microphone, mounted on or near the interaction table, recorded the speech of both child and examiner. Lapel microphones were wireless, so that participants were able to move freely around the room; in the case that the lapel microphone was distracting or bothersome for the child, the microphone was not used, and the boundary microphone was used instead to capture near-field speech of the child.

### Automated measure of latency

**Figure 2:**
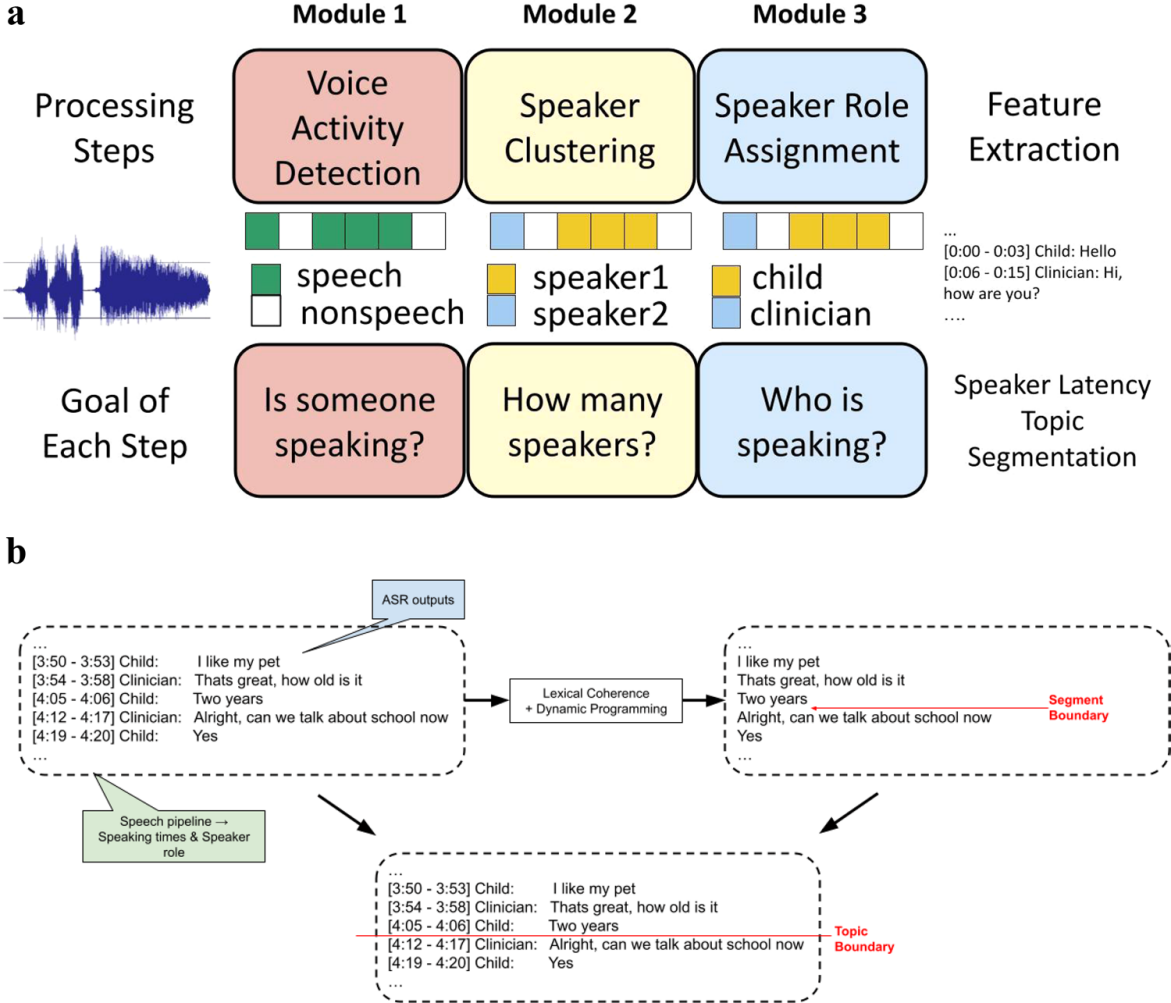
(**a**) Automated speech processing pipeline. Processing steps are depicted in the top row, with the goal of each step depicted in the bottom row. (**b**) Identification of topic boundaries. Topic boundaries (topic start and topic end) were computed automatically using the outputs from automatic speech recognition (ASR) system. Dynamic programming was used to divide the ASR transcript into contiguous segments.

We extracted conversational features for participants in each dyad using an automated speech processing pipeline. The pipeline consists of multiple modules that process the conversational audio to obtain speaking times for the child and the adult interviewer. Figure 2a depicts our novel technological approach.

For the first module, the speech activity detector, we used a two-hidden layer feed-forward deep neural network (DNN) to classify audio into speech and non-speech regions (background silence is treated as non-speech). The input consists of spliced (± 15 frames) 13-dimensional Mel frequency cepstral coefficients (MFCCs) extracted from the audio at each time frame to provide context, while the output nodes are binary labels (speech/non-speech). The DNN is trained to minimize cross-entropy loss. During testing, the class posteriors are smoothed and passed through a threshold to ascertain frame-level decisions about involving speech segments, or not. In the second module, audio speaker embeddings (x-vectors^44^) were computed at uniform intervals using the extracted speech segments. X-vector speech embeddings have been shown to capture speaker information from audio in multiple use-case scenarios^45,46^. Within each conversation, we clustered x-vectors into two clusters (child and adult) using agglomerative hierarchical clustering. At the final module, speaker roles (child and adult) were assigned to the two clusters based on the number of questions asked. For each cluster, an automatic speech recognition system (ASR) was used to convert the audio into text for obtaining a question count. The cluster with a higher number of questions was assigned as the adult, as expected in a natural social interaction between an adult and a child with ASD.

Once speaking roles (child vs. adult) were obtained for each participant, we computed latency as the duration (in seconds) of non-speech regions between speaker turns. We computed latency for the child and the examiner separately. As opposed to using the entire session to compute latency measures for the child and examiner, we restricted our computation to only times within a conversational topic. We defined a topic as a semantically coherent segment of conversation and estimated topic boundaries in an automated manner using the ASR outputs (text format). We segmented the ASR transcript for a BOSCC session into contiguous chunks (i.e., topics) using dynamic programming^47^ to optimize for the overall lexical coherence^48^ of the session that is computed as distance measures between word embedding representations. Combining this segmentation output with speaking times obtained from the speech processing pipeline, we obtained topic boundaries in the audio recording (see Fig. 2b). The topic boundaries allowed us to compute latency within each detected topic category, referred to as *intra-topic* latency. We were primarily interested in examining the time it takes for the child or examiner to respond when they are having back-and-forth exchanges *within* each conversational topic, since there is no clear expectation for social responses as one moves from one conversational topic to another.

### Data analysis

Data were analyzed using SPSS^®^ Statistics Version 26^49^. Pearson correlations were computed between intra-topic latency and baseline cognitive levels and autism symptom severity. Generalized Linear Mixed Models (GLMM) were used to determine whether significant improvements in conversational features were observed while controlling for baseline cognitive level, symptom severity, language level, age, and gender. GLMM allowed us to determine the effects of the predictor variables on the response variable while accounting for the repeated testing of the same participants (i.e., pre- to post-treatment). GLMM control for missing data^50^; therefore, we included cases with missing timepoints (n = 70 for participants with Time 1 data only). Mixed models included time, treatment condition, and the interaction between time and treatment condition as fixed effects. Additional covariates entered in each model as fixed effects included gender, baseline age, NVIQ, ADOS-2 CSS SA, and ADOS-2 expressive language level. Treatment duration and site effects were also added to secondary models to confirm that the results were not affected by confounding factors. Post-hoc paired sample *t* tests (two-tailed) were performed to examine the significant differences between T1 and T2 for each of the groups. Next, change scores for child and examiner intra-topic latency were examined using independent-samples *t* tests (two-tailed) across treatment and TAU groups. In addition, although independent samples *t* tests revealed that there were no significant differences in latency between treatment and TAU groups at Pre-Intervention (child latency, *t*[206] = − 0.915, *p* = 0.361, *d* = − 0.127; examiner latency, *t*[206] = − 1.17, *p* = 0.242, *d* = − 0.163), we conducted additional regression analyses to confirm there were still significant differences in latency between the treatment and TAU groups at Post-Intervention even after controlling for the Pre-Intervention latency as well as NVIQ, autism symptom severity, age, expressive language level, and gender.

## Data Availability

The data that support the findings of this study are available from the corresponding author (S.H.K.) upon reasonable request.
